# Video Demonstration of ABCA12‐Related Harlequin Ichthyosis in a Low‐Resource Setting: Case Report and Review of Early Management Challenges

**DOI:** 10.1002/ccr3.72633

**Published:** 2026-04-27

**Authors:** Chukwuka Elendu, Dependable C. Amaechi, Tochi C. Elendu, Emmanuel C. Amaechi, Ijeoma D. Elendu, Emeka H. Okolo, Mbanefo C. Uyanwune

**Affiliations:** ^1^ Federal University Teaching Hospital Owerri Nigeria; ^2^ Igbinedion University Benin City Nigeria; ^3^ Imo State University Owerri Nigeria; ^4^ Madonna University Elele Nigeria; ^5^ Gregory University Uturu Nigeria; ^6^ The Park Hospital Nottingham UK; ^7^ City St George's University of London London UK

**Keywords:** ABCA12, autosomal recessive congenital ichthyosis, harlequin ichthyosis, low‐resource settings, neonatal dermatologic emergency

## Abstract

Harlequin ichthyosis is a rare, life‐threatening neonatal dermatologic emergency that can be confidently diagnosed clinically at birth. Prompt recognition and early supportive management—including thermoregulation, fluid balance, infection prevention, and intensive skin care—are crucial determinants of survival, especially in low‐resource settings where diagnostic and therapeutic options are limited.

## 1 Introduction and Background

Harlequin ichthyosis is a rare and severe form of autosomal recessive congenital ichthyosis characterized by a markedly impaired epidermal barrier, resulting in thick, armor‐like hyperkeratotic plates and deep fissures at birth [1]. The condition is most commonly caused by pathogenic variants in the *ABCA12* gene, which encodes an ATP‐binding cassette transporter critical for lipid transport in lamellar granules and normal stratum corneum formation [2]. Defective lipid delivery leads to excessive transepidermal water loss, increased susceptibility to infection, respiratory compromise, and thermoregulatory instability, making it a neonatal dermatologic emergency [3].

Despite advances in neonatal intensive care and the use of systemic retinoids, mortality remains high, particularly in low‐resource settings where access to specialized care and supportive therapies is limited [4, 5]. This report presents a video‐demonstrated case of ABCA12‐related harlequin ichthyosis in a low‐resource setting, highlighting key clinical features and early management challenges relevant to similar contexts.

## 2 Case Description

The patient was a term neonate delivered at a peripheral health facility in a rural, low‐resource setting to a 27‐year‐old gravida 2 para 1 mother following an unbooked pregnancy. Antenatal care was minimal, with no documented prenatal ultrasounds or genetic screening. The mother reported no chronic illnesses, medication use, or teratogen exposure. There was no history of consanguinity or relevant family history of congenital skin disorders, neonatal deaths, or inherited conditions. Labor was spontaneous at an estimated 39 weeks' gestation, and delivery was vaginal without instrumental assistance. The infant cried weakly at birth, required brief stimulation, and had Apgar scores of 6 and 8 at one and 5 min, respectively.

**FIGURE 1:**
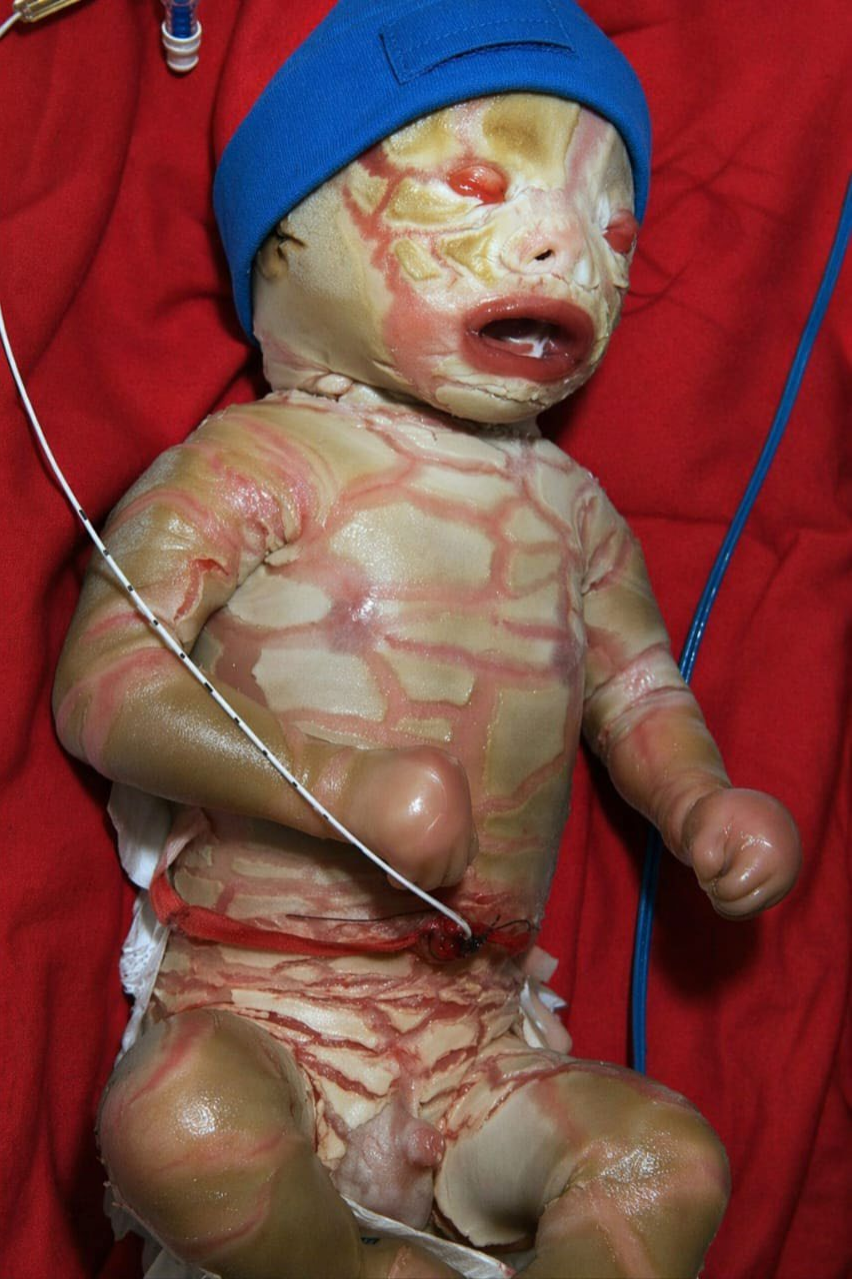
Representative still image from Video 1 showing a neonate with ABCA12‐related harlequin ichthyosis, demonstrating generalized thick hyperkeratotic plates with deep fissures, ectropion, and eclabium at presentation.

At birth, the neonate exhibited generalized thick, yellowish‐white hyperkeratotic plates separated by deep erythematous fissures involving the entire body surface (Figure 1). The rigid, taut skin markedly restricted limb movement and chest wall expansion. Facial features included severe ectropion with conjunctival exposure, eclabium with a persistently open mouth, flattened nasal alae, and poorly formed ears adherent to the scalp. The scalp showed extensive scaling. The extremities had flexion contractures with hypoplastic digits encased in hyperkeratotic plaques and circumferential fissures around the wrists and ankles. The palms and soles were thickened and fissured, limiting grasp and spontaneous movement. The hyperkeratotic plates showed variable prominence across different regions, likely reflecting early desquamation and changes in skin hydration over time. The umbilical cord and external genitalia were normal.

Within the first few hours of life, the neonate developed tachypnea, intermittent nasal flaring, and mild subcostal retractions, although oxygen saturation remained 95% on room air. Feeding was unsuccessful due to limited mouth opening and lip eversion. Deep fissures with serous ooze were noted in multiple areas without active bleeding. Vital signs revealed hypothermia (35.4°C), tachycardia (168 beats/min), and tachypnea (64 breaths/min). Weight was 3.1 kg (appropriate for gestational age), with mildly delayed capillary refill. Cardiovascular and abdominal examinations were unremarkable.

The infant was referred to a secondary‐level hospital for further care. During transport, thermal instability persisted due to lack of specialized neonatal transport equipment. On arrival, findings remained consistent with severe congenital ichthyosis, with diffuse hyperkeratotic plates, deep fissures, and minimal skin sparing. The conjunctivae appeared dry and erythematous with absent spontaneous blinking.

Basic laboratory investigations were limited by resource constraints. Random blood glucose and packed cell volume were within normal limits, while electrolyte and renal assessments were unavailable. No skin biopsy or genetic testing could be performed due to resource limitations. The diagnosis was made clinically based on the characteristic phenotype, consistent with harlequin ichthyosis and suggestive of an underlying ABCA12‐related disorder [2].

Over the next 24 h, the neonate developed worsening dehydration, persistent hypothermia, reduced urine output, and increased respiratory effort, likely exacerbated by restricted chest wall movement. Skin fissures deepened with progressive erythema, raising concern for secondary infection, although no overt signs of sepsis were observed. Feeding remained unsuccessful.

During hospitalization, there was minimal improvement in skin flexibility. Ectropion, eclabium, conjunctival dryness, and restricted limb movement persisted, with the infant remaining largely immobile. The characteristic clinical features are demonstrated in the accompanying video (Video 1), including generalized hyperkeratosis, facial abnormalities, limb contractures, and restricted chest wall expansion. The video was recorded using a handheld device under ambient lighting, reflecting documentation in a low‐resource setting.

**VIDEO 1:** Clinical video demonstrating generalized plate‐like hyperkeratosis, ectropion, eclabium, limb contractures, and restricted chest wall expansion in a term neonate with clinically diagnosed harlequin ichthyosis. Video content can be viewed at https://onlinelibrary.wiley.com/doi/10.1002/ccr3.72633.

Despite supportive care, the clinical condition remained fragile, with ongoing challenges in thermoregulation, fluid balance, and respiratory effort. This case highlights the reliance on clinical diagnosis and careful bedside assessment in resource‐limited settings.

### 2.1 Differential Diagnosis, Treatment Plan, and Follow‐Up

Although the clinical history and striking neonatal skin findings strongly suggested harlequin ichthyosis, alternative diagnoses were considered (Table 1), including collodion baby phenotypes and other severe neonatal dermatoses described in the literature [6, 7, 8, 9].

**TABLE 1:** Differential diagnosis of severe neonatal ichthyosis and distinguishing clinical features.

Condition	Typical neonatal presentation	Key distinguishing features	Rationale for exclusion in this case
Harlequin ichthyosis	Thick, plate‐like hyperkeratosis with deep fissures; severe ectropion and eclabium; limb constriction; restricted chest wall expansion	Most severe form of autosomal recessive congenital ichthyosis; profound skin rigidity and facial distortion; commonly associated with ABCA12 pathogenic variants	Findings fully consistent with observed phenotype
Collodion baby phenotype (lamellar ichthyosis/congenital ichthyosiform erythroderma)	Shiny, taut, translucent membrane at birth that desquamates over days to weeks	Lacks thick armor‐like plates, deep fissures, and severe ectropion/eclabium	Presentation showed rigid hyperkeratotic plates with deep fissuring rather than a collodion membrane
Epidermolytic ichthyosis	Neonatal erythema and blistering; skin fragility	Epidermal peeling and bullae predominate rather than massive hyperkeratosis	No blistering, skin fragility, or epidermal peeling observed
Restrictive dermopathy	Smooth, rigid, tight skin at birth; limited movement	Absence of fissures; often associated with pulmonary hypoplasia; distinct histopathology	Skin showed deep fissures and hyperkeratotic plates; respiratory findings consistent with chest wall restriction rather than pulmonary hypoplasia
Staphylococcal scalded skin syndrome	Acute onset erythema, skin tenderness, superficial epidermal peeling	Positive Nikolsky sign; systemic signs of infection	No epidermal sloughing, Nikolsky sign, or systemic sepsis at presentation

Management priorities centered on immediate supportive care to mitigate the life‐threatening consequences of profound epidermal barrier dysfunction, with interventions adapted to the constraints of a low‐resource setting (Table 2). Core measures such as thermoregulation, fluid and electrolyte monitoring, emollient therapy, and infection prevention are well established in the management of severe neonatal skin disorders [5, 7, 10]. In settings where available, systemic retinoids (e.g., acitretin) have been reported to accelerate desquamation and improve survival; however, access and monitoring remain limiting factors in low‐resource environments [11, 12].

**TABLE 2:** Acute management and follow‐up priorities in neonates with harlequin ichthyosis.

Care domain	Recommended interventions	Clinical rationale	Challenges in low‐resource settings
Thermoregulation	Thermoneutral care (incubator or warmed environment); avoidance of evaporative heat loss	Severe epidermal barrier disruption predisposes to hypothermia and metabolic instability	Limited availability of incubators; reliance on improvised warming methods
Fluid and electrolyte balance	Close monitoring of hydration status, urine output, and serum electrolytes; judicious fluid replacement	Excessive transepidermal water loss increases risk of dehydration and electrolyte disturbances	Limited access to laboratory testing and intravenous fluids
Skin care and barrier support	Liberal and frequent application of bland emollients (e.g., petrolatum‐based preparations); gentle handling	Improves skin flexibility, reduces fissuring, limits fluid loss, and enhances barrier function	Inconsistent supply of emollients; caregiver unfamiliarity with intensive skin care
Infection prevention	Strict aseptic handling; minimal skin trauma; early recognition and treatment of infection	Compromised skin integrity markedly increases susceptibility to bacterial sepsis	Limited availability of antibiotics and microbiologic testing
Ocular care	Regular ocular lubrication; protection of exposed conjunctivae	Severe ectropion predisposes to conjunctival dryness, keratitis, and infection	Limited access to ophthalmologic expertise and eye care supplies
Respiratory monitoring	Close observation of respiratory effort; supportive positioning; oxygen therapy if required	Rigid chest wall skin may restrict ventilation and contribute to respiratory compromise	Limited neonatal respiratory support equipment
Nutritional support	Early nutritional support (enteral or parenteral as feasible) to meet increased metabolic demands	Promotes growth, wound healing, and immune function	Feeding difficulties due to eclabium; limited nutritional supplementation options
Systemic retinoids	Early administration of acitretin where available, with laboratory monitoring	Accelerates shedding of hyperkeratotic plates and improves survival	Limited drug availability; inability to monitor hepatic function and lipids
Early follow‐up (neonatal period)	Serial assessments for dehydration, infection, electrolyte imbalance, and respiratory status	Mortality risk is highest in the first weeks of life	Fragmented follow‐up and delayed presentation
Long‐term multidisciplinary care	Dermatology, pediatrics, ophthalmology, nutrition, physiotherapy involvement	Addresses chronic hyperkeratosis, infections, contractures, growth failure, and ocular complications	Limited specialist access and referral pathways
Genetic counseling and family education	Counseling regarding autosomal recessive inheritance, recurrence risk, and prenatal options; caregiver education on skin care	Supports informed reproductive decision‐making and home‐based care	Limited access to genetic services and structured education programs

Follow‐up emphasized close monitoring in the early neonatal period, with ongoing assessment for complications such as dehydration, infection, and respiratory compromise [10]. Long‐term care often requires a multidisciplinary approach, alongside genetic counseling given the autosomal recessive inheritance pattern and recurrence risk [2, 6], with caregiver education remaining essential in resource‐limited settings.

## 3 Discussion

Harlequin ichthyosis represents the most severe end of the autosomal recessive congenital ichthyosis spectrum and is characterized by a striking and immediately recognizable neonatal phenotype. The clinical presentation in this case, characterized by generalized hyperkeratotic plates with deep fissuring and characteristic facial features, closely mirrors classic descriptions reported in large case series and consensus classifications of inherited ichthyoses [1, 5, 10]. These features, evident at birth and involving nearly the entire body surface, allow for confident clinical diagnosis even in the absence of genetic testing, which is particularly important in low‐resource settings where molecular diagnostics are often unavailable. The rigidity of the skin, restriction of spontaneous movements, and early physiological instability observed in this neonate underscore the severity of epidermal barrier dysfunction that defines harlequin ichthyosis and distinguishes it from milder collodion phenotypes or other disorders of cornification [6, 10].

At the molecular level, harlequin ichthyosis is most commonly caused by loss‐of‐function mutations in the ABCA12 gene, which encodes an ATP‐binding cassette transporter critical for lipid transport into lamellar granules within differentiating keratinocytes [2, 3]. Normal epidermal barrier formation depends on the secretion of these lipid‐rich granules into the extracellular space of the stratum corneum, where they organize into lamellar structures that prevent excessive transepidermal water loss and protect against environmental insults. In ABCA12 deficiency, impaired lipid transport leads to defective lamellar granule formation, abnormal stratum corneum lipid composition, and profound barrier failure, resulting in the massive hyperkeratosis and fissuring characteristic of harlequin ichthyosis [2, 3, 11]. The extreme phenotype observed in this case is consistent with previous reports demonstrating that truncating or null ABCA12 variants are associated with the most severe clinical manifestations and high early mortality [1, 11]. Although genetic confirmation was not possible, the constellation of findings strongly supports an ABCA12‐related etiology based on well‐established genotype–phenotype correlations [1, 10].

Respiratory compromise is a major contributor to early morbidity and mortality in harlequin ichthyosis and was a prominent concern in this case. The rigid, inelastic hyperkeratotic plates encasing the thorax mechanically restrict chest wall expansion, leading to shallow, rapid breathing and increased work of respiration despite structurally normal lungs [1, 10]. This mechanical limitation explains the early onset of tachypnea and respiratory distress observed in affected neonates. Video documentation in the present case provides important dynamic insight into disease severity by capturing the visibly restricted chest wall movement during respiration, a feature that is difficult to fully appreciate in static images alone. This real‐time visualization reinforces the underlying pathophysiologic mechanism of respiratory compromise and highlights the need for early respiratory monitoring even when oxygen saturation appears initially preserved [1, 10]. Beyond illustrating respiratory mechanics, the video also demonstrates the rigidity of the hyperkeratotic plates, depth of fissuring, limb immobility, and characteristic facial involvement, thereby complementing the written description and enhancing clinical recognition. Such visual documentation is particularly valuable for rare genodermatoses that may be encountered infrequently by clinicians, especially in low‐resource or peripheral settings where access to dermatology expertise is limited and may facilitate earlier diagnosis, appropriate triage, and informed counseling of caregivers [1, 10].

Management of harlequin ichthyosis is complex and resource‐intensive, focusing on mitigating the consequences of severe epidermal barrier failure rather than correcting the underlying genetic defect. Immediate priorities include supportive measures addressing thermoregulation, fluid balance, infection prevention, skin care, and other essential aspects of neonatal support [5, 10]. In high‐resource settings, advances in neonatal intensive care and the early use of systemic retinoids have significantly improved survival compared with historical outcomes [1, 4]. Retinoids such as acitretin accelerate desquamation of hyperkeratotic plates, improve skin flexibility, and may reduce complications related to constriction and barrier dysfunction [4, 12, 13]. However, their use requires careful dosing, monitoring for adverse effects, and access to laboratory testing, which may not be feasible in low‐resource environments. In the present case, limited availability of specialized neonatal care, lack of neonatal transport infrastructure, and absence of advanced monitoring constrained management options and necessitated reliance on pragmatic supportive measures achievable within existing resources.

The challenges highlighted in this case reflect broader systemic barriers to the care of rare congenital disorders in low‐resource settings. Delayed or absent antenatal care, lack of prenatal ultrasonography, and absence of genetic counseling limit opportunities for early diagnosis and perinatal planning. Postnatally, shortages of trained personnel, incubators, sterile supplies, and essential medications further exacerbate the risk of adverse outcomes. These constraints underscore the importance of clinical recognition based on phenotype alone and emphasize the need for simplified, evidence‐based management strategies that can be implemented in resource‐limited contexts [10]. Education of healthcare providers on the recognition and initial management of harlequin ichthyosis may help reduce preventable complications and improve early supportive care while awaiting referral where possible.

## 4 Concluding Remarks

Harlequin ichthyosis remains a life‐threatening neonatal condition in which outcomes depend largely on timely and adequate supportive care. In resource‐limited settings, careful clinical assessment and prompt implementation of essential interventions are critical for survival. The integration of video documentation alongside detailed clinical observation enhances recognition by illustrating disease severity, functional impairment, and dynamic features not fully captured by static images. Strengthening clinician awareness, improving referral pathways, and expanding access to essential neonatal care are key steps toward reducing preventable morbidity and mortality associated with this condition.

## Author Contributions

**Chukwuka Elendu:** conceptualization, investigation, project administration, supervision, validation, visualization, writing – original draft. **Dependable C. Amaechi:** data curation, methodology, writing – review and editing. **Tochi C. Elendu:** data curation, methodology, resources, writing – review and editing. **Emmanuel C. Amaechi:** formal analysis, validation, writing – review and editing. **Ijeoma D. Elendu:** visualization, writing – review and editing. **Emeka H. Okolo:** supervision, writing – review and editing. **Mbanefo C. Uyanwune:** investigation, writing – review and editing.

## Funding

The authors have nothing to report.

## Consent

Written informed consent was obtained from the patient's parent/legal guardian for publication of this report and the accompanying clinical video.

## Conflicts of Interest

The views expressed in this report are solely those of the authors and do not represent the official positions of any affiliated institutions. The authors declare no conflicts of interest and received no funding for this work. Ethical approval was not required, in accordance with the policies of the affiliated institution.

## Data Availability

The authors have nothing to report.
